# Metabolite secretions of *Lactobacillus plantarum* YYC-3 may inhibit colon cancer cell metastasis by suppressing the VEGF-MMP2/9 signaling pathway

**DOI:** 10.1186/s12934-020-01466-2

**Published:** 2020-11-23

**Authors:** Yuan-Chun Yue, Bao-Yu Yang, Jing Lu, Shu-Wen Zhang, Liu Liu, Khaled Nassar, Xiao-Xi Xu, Xiao-Yang Pang, Jia-Ping Lv

**Affiliations:** 1 Institute of Food Science and Technology, Chinese Academy of Agricultural Sciences, Beijing, 100193 People’s Republic of China; 2 College of Food Science, Northeast Agricultural University, Harbin, 150030 People’s Republic of China

**Keywords:** *Lactobacillus plantarum*, Metastasis, Colon cancer cell, VEGFA

## Abstract

**Background:**

Colorectal cancer (CRC) is a major clinical challenge, and the gut microbiome plays important roles in the occurrence and metastasis of CRC. *Lactobacillus* and their metabolites are thought to be able to suppress the growth of CRC cells. However, the antimetastatic mechanism of *Lactobacillus* or their metabolites toward CRC cells is not clear. Therefore, the aim of this study was to assess the inhibitory mechanism of cell-free supernatants (CFSs) of *L. rhamnosus* GG, *L. casei* M3, and *L. plantarum* YYC-3 on metastasis of CRC cells.

**Results:**

YYC-3 CFS showed the highest inhibitory effect on CRC cell growth, invasion and migration, and inhibited MMP2, MMP9, and VEGFA gene and protein expression, and protein secretion. Furthermore, it suppressed the activities of MMPs by gelatin zymography. Moreover, the effective compounds in these CFSs were analyzed by Q Exactive Focus liquid chromatography–mass spectrometry.

**Conclusions:**

Our results showed that metabolite secretions of YYC-3 may inhibited cell metastasis by downregulating the VEGF/MMPs signaling pathway. These data suggest that treatment of CRC cells with metabolites from *L. plantarum* YYC-3 may reduce colon cancer metastasis.

## Background

Colorectal cancer (CRC) is a major cause of cancer-related mortality worldwide, and many patients with CRC are in an advanced stage with distant metastasis [1–3]. The main inducing factors of colon cancer are genes and the gut environment. The gut microbiome plays a critical role in maintaining the health of the gastrointestinal tract through host-microbe and microbe–microbe interactions [4–6]. Studies on the impact of the gut microbiota and the effects its metabolite secretions on proliferation and metastasis of colon cancer cells may provide new strategies for treatment of colon cancer. Previous studies have reported that gut microbiota have an effect on the progress of CRC, such as *Fusobacterium nucleatum* which has a symbiotic relationship with colon cancer and encourages proliferation of colon cancer cells [7].

Lactic acid bacteria (LAB), such as *Lactobacillus* and *Bifidobacterium*, are also members of the gut microbiome [8–11]. Some metabolites secreted by LAB exhibit antibacterial properties, for example, bacteriocin; these metabolites may be potent substitutes for antibiotics [12, 13].

In recent years, the applications of LAB have extended into the field of cancer prevention, especially for CRC. Previous reports have demonstrated that certain LAB, including *Lactobacillus casei*, *L. paracasei*, *L. plantarum*, and *L. reuteri*, can inhibit the growth of cancer cells [14–16]. The anti-cancer mechanisms attributed to these LABs include promoting apoptosis of the cancer cells through intrinsic and extrinsic apoptotic pathways, anti-proliferative regulation of the cell cycle in cancer cell lines, sequestering of reactive oxygen species by antioxidative enzymes to prevent carcinogenesis, production of bacterial enzymes, effecting the metabolites and epigenetics of the host, and regulating different signalling pathways in colon cancer cells [17, 18].

Vascular endothelial growth factor (VEGF)-MMPs is one of the most important signalling pathways and is involved in multiple cellular processes, including cell migration, invasion, and vascular cell permeability [19]. VEGF is considered the major endothelial mitogen in the neoplasms of the central nervous system [20]. In some tumours, the expression of VEGFA is associated with angiogenesis and metastasis, which are processes involved in the invasion of cancer cells using the extracellular matrix (ECM). In the VEGF-MMPs signalling pathway, VEGF binds to its receptor, VEGFR, which is associated with the secretion of the downstream target, matrix metalloproteinases (MMPs). MMPs are calcium-dependent and zinc-containing endopeptidases that can be induced by an autocrine VEGF/VEGFR signalling pathway loop [21]. It had been reported that cancer cells use MMPs to degrade ECM during invasion and metastasis [22].

Previously, we identified *L*. *rhamnosus* GG, *L*. *casei* M3 and *L*. *plantarum* YYC-3 from 120 strains of LAB by the high antibacterial activities of their cell free supernatants (CFSs) using the cup-plate method on *F. nucleatum,* which is the symbiotic strain involved in colon cancer (not published)*.* We found that *L*. *plantarum* YYC-3 modulated the tumour microenvironment to prevent colon cancer in *APC*^*Min/*+^ model mice [23]. However, the direct effects of *L*. *plantarum* YYC-3 CFS on human colon cancer cell metastasis remains unclear.

Therefore, the aim of this study was to investigate the inhibitory effects of the metabolite secretions of *L*. *rhamnosus* GG, *L*. *casei* M3 and *L*. *plantarum* YYC-3 on colon cancer cell metastasis, and to determine their molecular mechanisms using the human colorectal carcinoma cell lines Caco-2 and HT-29.

## Results

### Cytotoxicity of colon cancer cells treated with cell free supernatants from *Lactobacillus*

To explore the effect of *Lactobacillus* CFS on colon cancer cytotoxicity (Table 1), the CCK-8 method was used. In both the Caco-2 and HT-29 cells, an increasing concentration of *Lactobacillus* CFS resulted in enhanced cytotoxicity. At a concentration of 800 µL/mL, the cell viability was inhibited the most effectively (as high as 100%) in Caco-2 cells treated with CFS of M3 (Table 1). The half maximal inhibitory concentration (IC_50_) values of all the treatment groups (GG, M3, and YYC-3) were 344.81, 291.66, and 312.78 μL/mL, respectively. Similarly, the growth of the HT-29 cells were also inhibited in a dose-dependent manner after treatment with CFSs of GG, M3, and YYC-3, the IC_50_ values of these were 358.21, 349.88, and 259.91 μL/mL, respectively. Their inhibitory rates were comparable to those of 2.5 μM 5-flourouracil (5-FU, positive control) which could inhibit approximately 50% of the HT-29 cells. Treatment with MRS medium showed no significant reduction in the viability of the Caco-2 and HT-29 cells. These results indicate that the CFSs of YYC-3 has the highest cytotoxicity effect among the three LABs on colon cancer cells.

**Table 1:** The IC_50_ value (μL/mL) by CFSs of LAB on Caco-2 and HT-29 cells

Concentration (μL/mL)										
Cell lines	Strains	80	100	200	300	400	500	600	700	IC_50_ value
Caco-2	GG	–	1.63 ± 0.03	22.83 ± 0.19	40.28 ± 0.13	53.76 ± 0.04	61.62 ± 0.08	84.53 ± 0.15	92.22 ± 0.06	344.81 ± 0.08
	M3	–	7.28 ± 0.06	27.52 ± 0.13	51.16 ± 0.09	68.30 ± 0.12	72.83 ± 0.04	89.32 ± 0.11	96.48 ± 0.13	291.66 ± 0.13
	YYC-3	–	5.27 ± 0.01	26.39 ± 0.06	48.23 ± 0.16	60.27 ± 0.08	69.44 ± 0.11	88.10 ± 0.13	94.72 ± 0.09	312.78 ± 0.06
	MRS	–	–	–	–	–	–	–	0.02 ± 0.001	–
HT-29	GG	–	3.41 ± 0.03	12.75 ± 0.22	39.47 ± 0.12	50.11 ± 0.16	73.59 ± 0.13	82.93 ± 0.09	95.37 ± 0.12	358.21 ± 0.08
	M3	–	3.16 ± 0.01	10.73 ± 0.08	43.21 ± 0.12	50.09 ± 0.11	75.28 ± 0.18	86.81 ± 0.11	92.42 ± 0.17	349.88 ± 0.21
	YYC-3	–	9.77 ± 0.01	30.69 ± 0.10	63.73 ± 0.08	72.71 ± 0.13	80.10 ± 0.02	89.73 ± 0.07	93.58 ± 0.06	259.91 ± 0.10
	MRS	–	–	–	–	–	–	–	–	–

CCK8 was used to carried out and using Logit of probit regression in SPSS19.0

### Analysis of cell invasion and cell migration

**Fig. 1:**
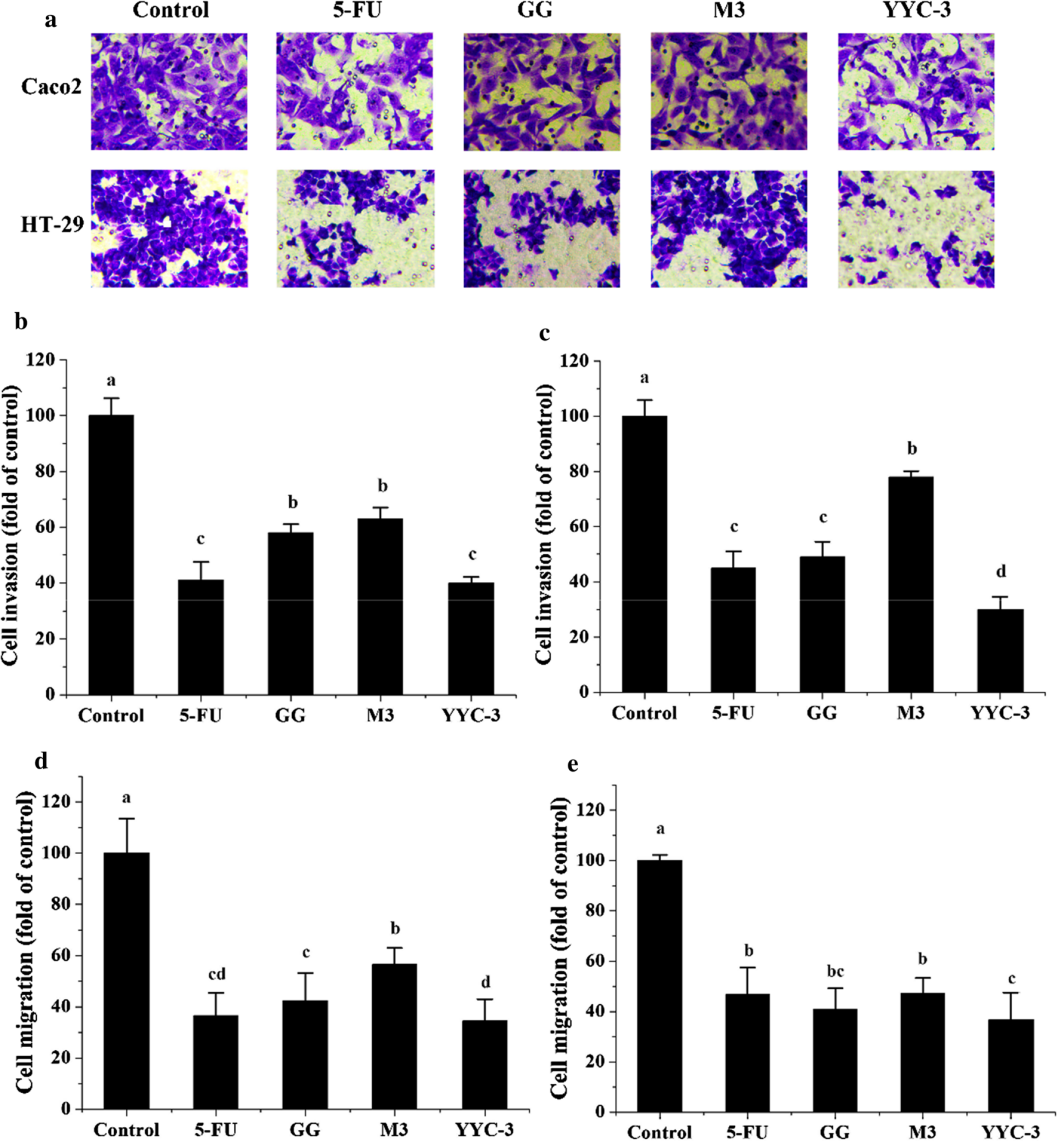
*Lactobacillus* CFSs inhibit Transwell™ invasion and migration of colon cancer cells. **a** The images of cell invasion in Caco-2 and HT-29. **b** Cell invasion of Caco-2; **c** Cell invasion of HT-29; **d** Cell migration of Caco-2; **e** Cell migration of HT-29. All CFS treatments inhibited Transwell invasion and migration compared with negative controls at the concentration of 80 μL/mL (untreated group is the negative control and the 5-FU treatment group is the positive control; error bars represent mean of each group ± SD of three independent experiments; a–d different letters in bars mean P < 0.05 (significant differences) by One-way ANOVA in the same group)

To analyse the effect of *Lactobacillus* CFS on colon cancer cell metastasis, the invasion and migration of colon cancer cells was evaluated with Transwell™ assays. These results are shown in Fig. 1, treatment with CFS limited the ability of the Caco-2 and HT-29 cells to traverse and invade the filter membranes as shown in Fig. 1a, b, and suppressed cell migration (Fig. 1c, d, *P* < 0.05). For Caco-2 cells, treatment with the CFS of YYC-3 showed the highest inhibition of cell invasion. Compared with the negative control (90 ± 4.3 cells), only 40% of cells (36 ± 2.1 cells) in the treated group of YYC-3 traversed through the filter membranes, which was significantly fewer than the control (*P* < 0.05). The migration of Caco-2 cells was also significantly inhibited with the treatment of the CFS of YYC-3, with only 34% of cells (56 ± 1.8 cells) traversing through the filter membranes (data in Fig. 1 and the images shown in Additional file 1: Figure S1). Moreover, the other two groups also exhibited significantly limited cellular invasion and migration. Among the three treatment groups of CFS, the cells with YYC-3 showed the highest ability to inhibit the invasion and migration of Caco-2 and HT-29 cells.

### Analysis of the effective compounds in the CFS

**Fig. 2:**
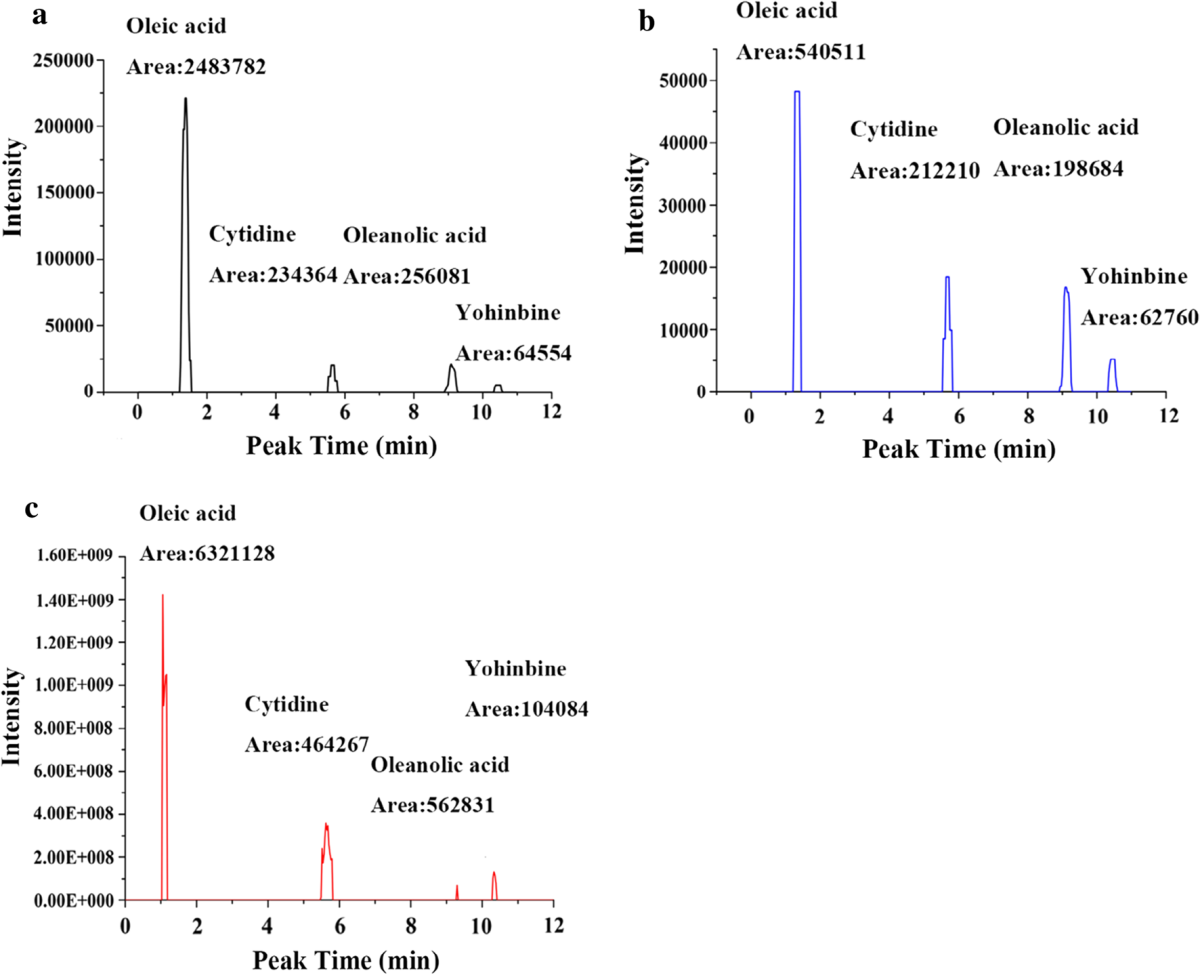
Inhibitory effect of CFSs on cell growth and effective compound analysis of CFSs. **a** Cell growth of Caco-2; **b** Cell growth of HT-29; **c** Effective compound analysis of YYC-3 CFSs. Using the method of CCK-8, and 10% (v/v) CFSs were added in the treated groups and cultured for 24, 48, and 72 h, respectively. And (UPLC)-Q Exactive Focus-MS/MS was used for evaluated effective compounds. Treatment groups and positive control were compared with the negative control, and * means *P* < 0.05, **means *P* < 0.01, ***means *P* < 0.001

To identify the potential effective compounds by which CFSs affect colon cancer cell metastasis, Q Exactive Focus liquid chromatography-mass spectrometry was used to analyse the metabolites in these LABs. Figure 2 shows the four identified effective compounds, which included oleic acid, cytidine, oleanolic acid, and yohimbine. The molecular weights in the database were 522.36, 243.09, 456.36, and 354.19, respectively. The production of these four compounds was highest in the YYC-3 strain, followed by GG then M3. Compared with the other three compounds, the output of oleic acid was the highest. These results indicate that oleic acid, cytidine, oleanolic acid, and yohimbine are the potential effective compounds in the CFSs that inhibit colon cancer cell metastasis.

### Analysis of gene expression

**Fig. 3:**
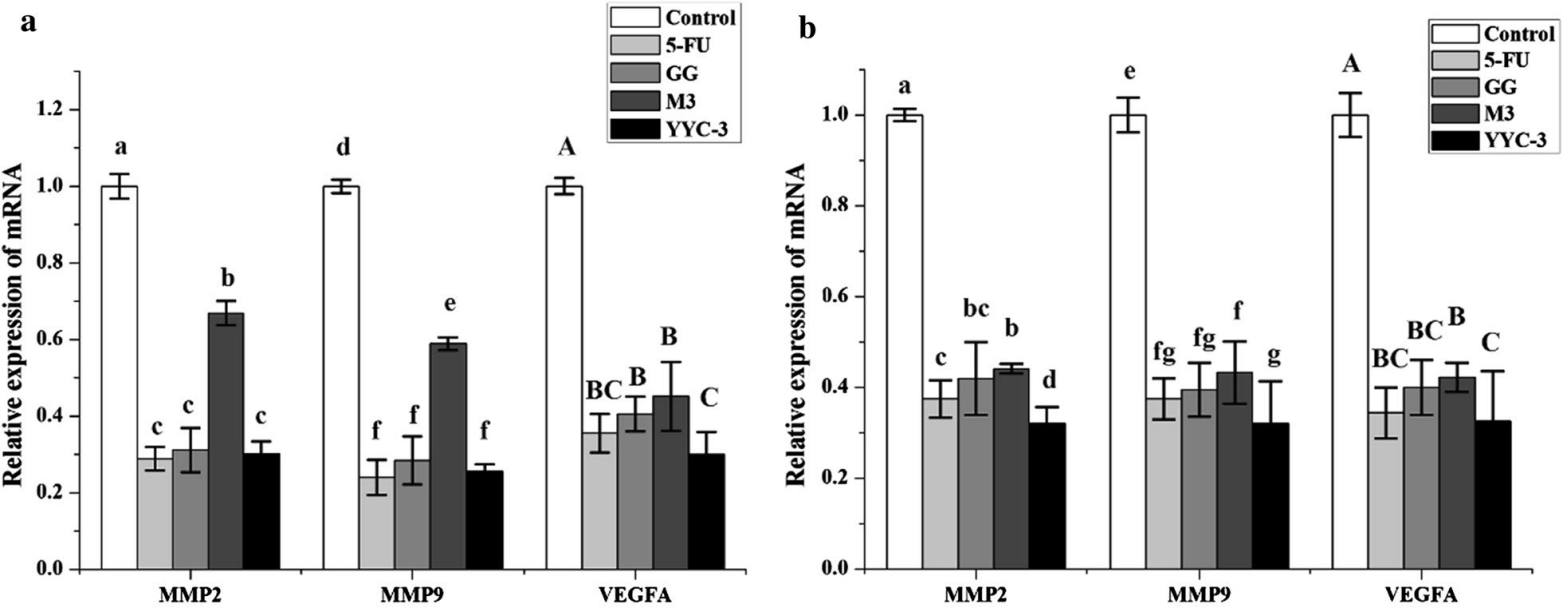
Relative gene expression of metastasis-related genes in colon cancer cells treated with Lactobacillus CFS. **a** Gene expression in Caco-2 cells; **b** Gene expression in HT-29 cells. Real-time PCR was carried out to assess changes in MMP2, MMP9, and VEGFA gene expression in colon cancer cells treated with Lactobacillus CFS (untreated group is the negative control and the 5-FU treatment group is the positive control; error bars represent mean of each group ± SD of three independent experiments; a–d, e–g, A–C, different letters in bars mean *P* < 0.05 (significant differences) by One-way ANOVA in the same group)

In view of the mechanism by which these identified compounds in the CFSs exert their effect on cancer cell metastasis, by inhibiting VEGF. We further verified the effective compounds, by analysing the key genes *MMP2*, *MMP9*, and *VEGFA* in the VEGF-MMPs signalling pathway of colon cancer cells using real-time (RT)-PCR (Fig. 3). Compared with the negative control, the expression of *MMP2*, *MMP9*, and *VEGFA* were significantly decreased in all the CFS-treated groups. In Caco-2 cells, the YYC-3 treated group had significantly reduced *MMP2*, *MMP9*, and *VEGFA* gene expressions that were 0.30, 0.25, and 0.30 fold of the negative control group, respectively. The other two treatment groups also had reduced *MMP2*, *MMP9*, and *VEGFA* gene expression. In HT-29 cells, these three gene expressions were significantly reduced in the YYC-3 treated groups compared with the negative control group (~ 0.32 fold). Moreover, treatment with YYC-3 CFS resulted in the largest reduction of these gene expressions, about 0.3-fold compared with the negative control group.

### Analysis of protein levels

**Fig. 4:**
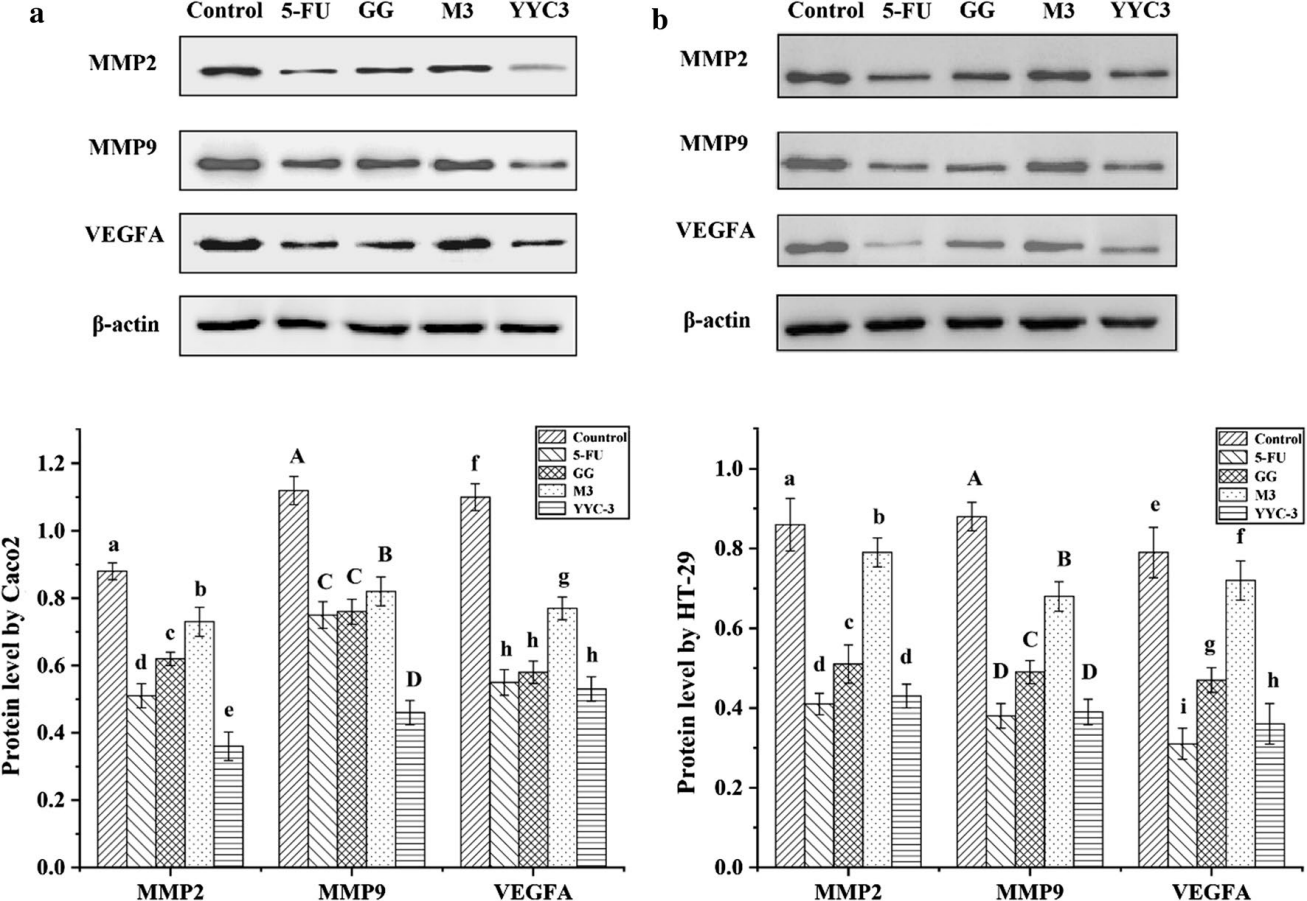
Relative protein level from metastasis-related genes in colon cancer cells treated with Lactobacillus CFS. **a** Protein level of Caco-2; **b** Protein level of HT-29. Western blot analysis was performed to determine changes in protein level of MMP2, MMP9, and VEGFA in colon cancer cells treated with Lactobacillus CFS (untreated group is the negative control and the 5-FU treatment group is the positive control; error bars represent mean of each group ± SD of three independent experiments; a–e, A–D, f–i, different letters in bars mean P < 0.05 (significant differences) by One-way ANOVA in the same group)

Western blots were used to survey the inhibitory ability of Lactobacillus CFS on the protein levels of MMP2, MMP9, and VEGFA in colon cancer cells. Treatment with YYC-3 resulted in the greatest inhibition of MMP2, MMP9, and VEGFA protein levels in both the Caco-2 and HT-29 cells. Moreover, treatment with M3 CFS repressed MMP2 and VEGF protein levels in Caco-2 cells, but did not induce a significant reduction (P > 0.05) in MMP9 protein levels (Fig. 4a). Treatment of HT-29 cells with M3 CFS, however, did result in a significant decrease in the levels of MMP2, MMP9, and VEGFA (Fig. 4b).

### Assessment of protein secretion

**Fig. 5:**
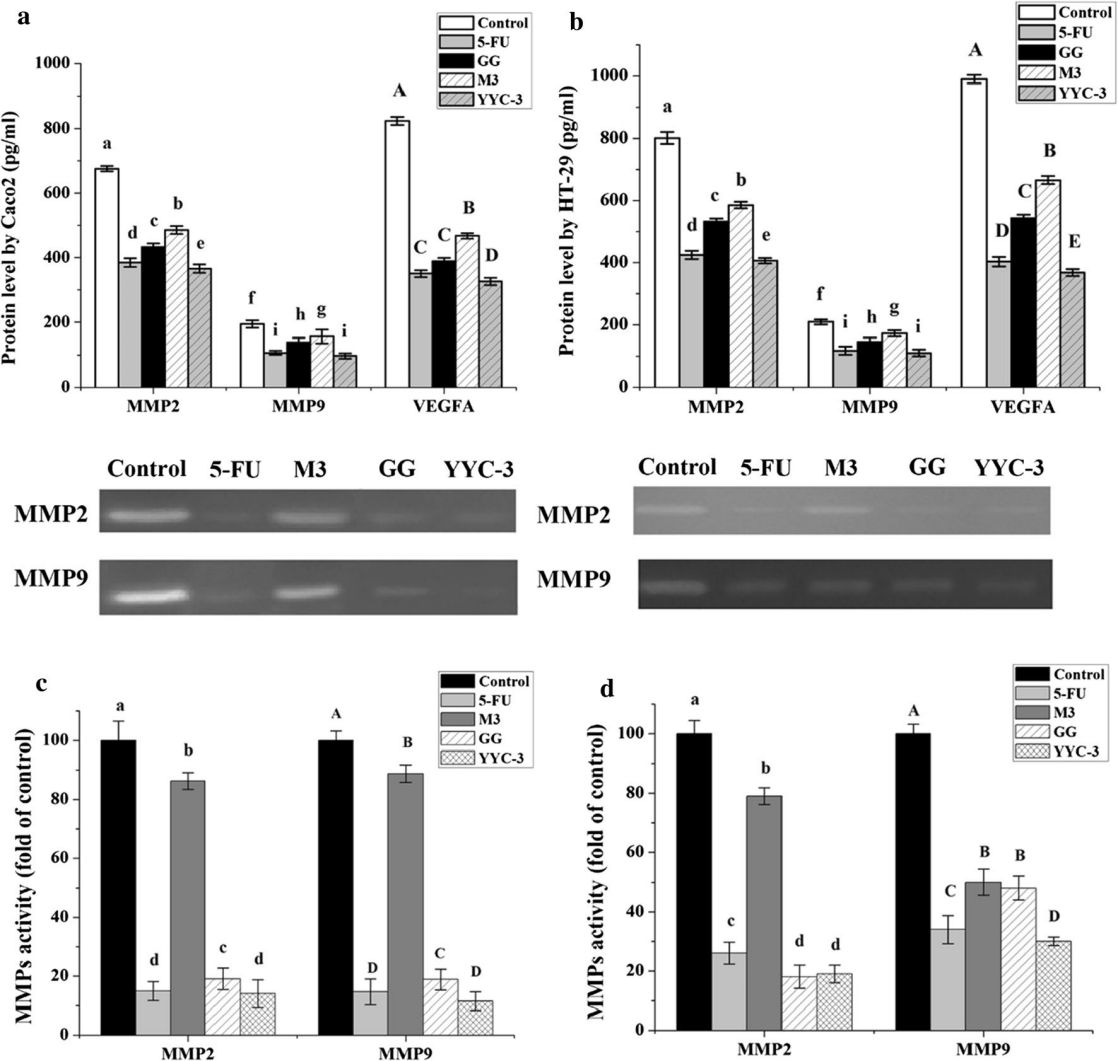
Protein secretion of MMP2, MMP9, and VEGFA and activity of MMPs by colon cancer cells treated with Lactobacillus CFS. **a** Caco-2 protein secretion; **b** HT-29 protein secretion; **c** Caco-2 MMPs activity. **d** HT-29 MMPs activity. ELISA analysis was performed to determine changes in protein secretion of MMP2, MMP9, and VEGFA and gelatin zymography was used to test the activity of MMPs in colon cancer cells treated with Lactobacillus CFS (untreated group is the negative control and the 5-FU treatment group is the positive control; error bars represent mean of each group ± SD of three independent experiments; a–e, f–i, A–E, different letters in bars mean *P* < 0.05 (significant differences) by One-way ANOVA in the same group

VEGF-MMPs are involved in the degradation of essentially all extracellular matrix (ECM) components but they must be secreted into the extracellular matrix for this action. Thus, the level of secreted VEGF-MMPs proteins were determined. The secretion of MMP2, MMP9, and VEGFA proteins in the YYC-3 treated group were less than those of the other groups (Fig. 5a, b). The treatment of Caco2 cell with YYC-3 CFS showed that MMP2, MMP9, and VEGFA (360.8, 96.9, and 326.4 pg/mL, respectively) were significantly lower than the untreated group (*P* < 0.001). However, in HT-29 cell, YYC-3 CFS treatment resulted in a protein secretion level of 406.1, 109.5, and 367.5 pg/mL in these proteins, respectively, which was also significantly lower than the untreated group (*P* < 0.001). Furthermore, when the YYC-3 treated group was compared with the positive control (5-FU), there was a significant difference (*P* < 0.05) in the MMP2 and VEGFA protein secretion levels; although no significant differences (*P* > 0.05) was observed in the MMP9 was observed in both the Caco2 and HT-29 cells.

### MMP2 and MMP9 activities by gelatine zymography

To test the inhibitory ability of Lactobacillus CFSs on the activity of MMP-2 and MMP-9. The activities of MMP2 and MMP9 were evaluated using gelatine zymography. The results are shown in Fig. 5c, d. All the treatment groups showed significant inhibition of MMP2 and MMP9 activity in both the Caco2 and HT-29 cells (Fig. 5c, *P* < 0.05). Among them, the YYC-3 treated group showed the highest ability to suppress MMP2 and MMP9 activities, which decreased to 19.1 ± 2.9% (MMP2) and 30.3 ± 1.4% (MMP9) compared to the control in Caco2 cells, respectively. Moreover, for HT-29 cells, YYC-3 downregulated MMP2 and MMP9 activity to 14.2 ± 4.7% (MMP2) and 11.5 ± 3.1% (MMP9) compared to the control, respectively.

## Discussion

LAB are probiotic microorganisms that have been implicated as anticancer agents, and may inhibit colon cancer cells through direct interactions and/or via the secretion of bacteriocin and other bioactive components [24–26]. P8 protein that produced by *L. rhamnosus* KCTC 12202BP, shows strong inhibitory effect on colon cancer cell by inducing cell cycle arrest [27]. And Lee et al. evidenced the anti-cancer activity of the probiotic bacterium *L. fermentum* using 3D culture in colon cancer cells [28]. Moreover, Carmen et al. revealed LAB shows anti-cancer effect by expressing antioxidant enzymes or IL-10 in colon cancer model mice [29]. However, only a few reports have focused on the effects of LAB and their metabolites on colon cancer cell metastasis. In this study, we used the metabolites of three LAB (*L*. *rhamnosus* GG, *L*. *casei* M3 and *L*. *plantarum* YYC-3) to explore their anticancer effects and the related molecular mechanisms on colon cancer cell lines (Caco-2 and HT-29). We found that the metabolites of these LABs could suppress the metastasis of colon cancer cells by inhibiting the VEGF/MMPs signalling pathway, which demonstrated the anticancer function of these LAB.

LAB and its metabolite secretions have been evidenced to be cytotoxic to colon cancer cells. Faghfoori et al. demonstrated that *L. plantarum* had a cytotoxic effect on HT-29 cells [30]. Our results showed that the CFS of YYC-3 had the highest inhibitory activity toward HT-29 cell, with an IC_50_ value of 259.91 ± 0.10 μL/mL. Similarly to the results of Chen et al. [31], who also found that *L. plantarum* PM153 exhibited an anticancer effect on HT-29 cell (IC_50_ value was 299.3 μL/mL) under similar conditions. However, Vemuri et al. [32] found that *L. plantarum* UALp-05 did not significantly influence on HT-29 cells. These results suggest that the inhibitory activity by *L. plantarum* may be strain-specific.

Cancer-associated mortality is mainly associated with metastasis from the primary tumour to distant organs, such as lung, liver, and brain [33]. For the anti-metastasis of LAB on colon cancer cells, Escamilla et al. [34] reported that the CFSs of *L. casei* and *L. rhamnosus* GG had inhibitory effects on the invasion of HCT-116 colon cancer cells. They also reported that the CFS of *L. casei* showed a higher inhibitory effect on the cell invasion than that of *L. rhamnosus* GG. In this study, we also demonstrated the inhibitory effect of CFSs by *L. casei* and *L. rhamnosus* GG on colon cancer cell invasion and migration using Caco-2 and HT-29 cells. Contrary to the report of Escamilla et al., we found that CFS of *L. casei* M3 had a lower inhibitory effect on colon cancer cell metastasis when compared with that of *L. rhamnosus* GG. The variations of our report with that of Escamilla et al. could be strain-dependent or due to cell line differentiation.

In this study, all treatment groups had significant inhibitory effects on Caco2 and HT-29 cell invasion and migration. However, the YYC-3-treated group showed the highest ability to inhibit the invasion and migration of the cells, when compared with the other treatment groups. Therefore, it could be inferred that the CFS of YYC-3 may have the most effective compounds compared to the other two stains.

There are many reports on the inhibitory effect of natural products in tumour metastasis. To evaluate the specific compounds implicated in the inhibitory activity, the CFSs of the three strains were analysed using Q Exactive Focus liquid chromatography–mass spectrometry, and oleic acid, oleanolic acid, yohimbine and cytidine were identified in all three CFSs. The concentrations of these substances positively correlated with the anticancer activity demonstrated by the strains. Interestingly, several reports have proven that oleic acid, oleanolic acid, yohimbine and cytidine possess anticancer activity by the suppressing tumour metastasis [35–38]. Furthermore, natural products containing these compounds have been reported to inhibit the expression of VEGF. This is an angiogenic factor, and is closely related to organ development, endothelial cell growth, and blood vessel permeability [39]. VEGFA also facilitates the invasion of cancer cells via the VEGFA/VEGFAR pathway by activating p38 mitogen-activated protein kinase MAPK and phosphatidylkinase B (AKT) in order to induce MMP expression, which are widely used to indicate the metastatic capability of colon cancer cells [21]. MMP2 and MMP9 are gelatinases that are able to degrade and remodel ECM [20]; for these reasons MMP2 and MMP9 play important roles in promoting the metastasis of tumour cells. Furthermore, the effect of CFSs from LAB on gene expression, protein levels and protein secretion in the colon cancer cell experiments have verified this molecular mechanism, which is the inhibition of the VEGF-MMP2/9 pathway and evidence the effective compounds of oleic acid, oleanolic acid, yohimbine and cytidine.

The inhibition of MMP2 and MMP9 could suppress the metastasis of cancer cells. The CFSs of *L. rhamnosus* GG and *L. crispatus* have been reported to downregulate MMP2 and MMP9 in HT-29 and HeLa cells, thus indicating anti-metastasis [40]. Moreover, Norouzi et al. [41] reported that nisin, as the product of *Streptococcus lactate*, could attenuates the expression of the metastatic genes MMP2 and CEA inhibiting colon cancer cell metastasis. In this study, we demonstrated that the metabolite secretions of GG, M3 and YYC-3 suppressed VEGFA expression and secretion, and limited the expression and secretion levels of MMP2 and MMP9; suggesting that the inhibition of VEGF-MMP signalling pathway is suppressing cancer cell metastasis. Similarly, the YYC-3 treatment group exhibited the highest inhibitory activity on the target gene protein levels and secretions in Caco-2 and HT-29 cells. These results further validated the highest inhibitory activity demonstrated on cell invasion and migration of Caco-2 and HT-29 cells by YYC-3. However, Escamilla et al. [34] showed that the CFS of *B. thetaiotaomicron* did not inhibit colon cancer cell invasion.

**Fig. 6:**
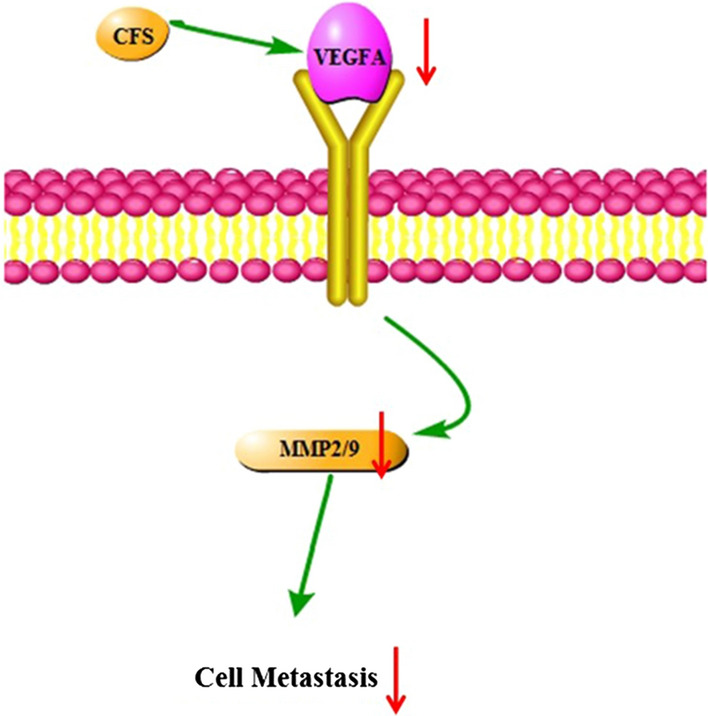
Putative mechanism by which metabolite secretions of *L. plantarum* YYC-3 inhibit colon cancer cell viability and metastasis

The pathway by which the CFSs of YYC-3, M3 and GG inhibit colon cancer cell metastasis are modelled in Fig. 6. The pathway indicated that these CFSs inhibited the expression of VEGFA, thereby limiting the VEGFA/VEGFAR pathway. It also showed that the expression of MMP2 and MMP9 were suppressed, which resulted in the inhibition of colon cancer metastasis.

## Conclusion

In this paper, we demonstrate the inhibition of cell growth and antimetastatic activities of the metabolite secretions of *L. rhamnosus* GG, *L. casei* M3, and *L. plantarum* YYC-3 toward colon cancer cell lines. The inhibitory activities of these CFSs were as effective as that of a conventional chemotherapy drug (5-FU). Among these *Lactobacillus* strains, *L. plantarum* YYC-3 showed the best inhibitory activities against the metastasis of colon cancer cells. These CFSs suppress VEGFA expression, and may exert their anticancer activities through repression of MMP2 and MMP9 downstream of the VEGFA/VEGFAR pathway. This study demonstrates that these three *Lactobacillus* strains may have potential as alternatives to conventional drugs for the treatment of colon cancer. Further research is required to explore the effects of these probiotic bacteria on colon cancer cells, as well as to assess the inhibitory activities of these bacterial cells and their supernatants in vivo.

## Methods

### Cell lines and culture conditions

Human colon cancer cell lines (HT-29 and Caco-2) were purchased from Procell Life Science & Technology Co., Ltd., China. HT-29 cell was cultured in Dulbecco’s modified Eagle’s medium (DMEM)/high glucose medium (Procell Life Science & Technology Co., Ltd.) supplemented with 10% foetal bovine serum (FBS) and Caco2 cell was cultured in minimum Eagle’s medium (MEM) with 20% FBS, both of them contains 1% penicillin (100 IU/mL) and streptomycin (100 μg/mL) in sterile conditions at 37 °C in a humidified atmosphere with 5% CO_2_. The culture medium was replaced every 2 days.

### Bacterial culture and preparation of cell-free supernatant

*Lactobacillus rhamnosus* GG and *L. casei* M3 were obtained from the Institute of Food Science and Technology, Chinese Academy of Agricultural Sciences (CAAS), Beijing, China. *L. plantarum* YYC-3 was isolated from fermented rose juice, as previously described [23]. All the strains obtained were cultured using 5% inoculum size in 100 mL MRS broth, and then incubated anaerobically in an anaerobic jar (UNITECH, UT706, China) together with GENbag anaer (AnaeroGen, Oxoid) at 37 °C without shaking for 24 h. Thereafter, 1 mL of these cultures, containing about 10^9^ colony-forming units/mL of LAB, was centrifuged at 6010×*g* for 10 min and the supernatants were retained. The pH of the supernatants was adjusted to 7.2 using 1 M NaOH, and then the supernatants were passed through 0.22 μm filters (Sartorius Stedim Biotech). The filtrates obtained were used for analysis. 2.5 μM (Selleck, USA) of 5-FU was used as positive control in this manuscript.

### Analysis of cytotoxicity

Cell Counting Kit-8 (CCK-8) (Solarbio, China) assays were used to evaluate the cytotoxic impact of *Lactobacillus* CFS on CRC cells. Briefly, 5 × 10^4^ CRC cells were seeded into each well of a 96-well plate in 100 μL complete medium. Cells were incubated overnight in normal cell growth conditions to enable cells to adhere to the wells. Next, the medium in each well was replaced, and different concentrations of *Lactobacillus* CFS (20, 30, 40, 50, 60, 70, 80, and 100% CFS, diluted with complete medium) were added and cultured for 24 h to test cytotoxicity effect and measure the IC_50_ value. Next, 10 μL of CCK-8 solution was added to each well and the plates were incubated at 37 °C for an additional 4 h, and, 100 μL complete medium were used as negative controls; 2.5 μM 5-FU was used as a positive control for cytotoxicity. After 24 h incubation, 10 μL of CCK-8 solution were added to each well and the plates were incubated at 37 °C for an additional 4 h. Cytotoxicity effect was measured using a spectrophotometer (TECAN, Spark 20 M, Switzerland) at 450 nm. Inhibition ratio (%) = [(OD_untreated_ – OD_treated_)/(OD_untreated_)] × 100%. The analysis was performed in triplicate. The IC_50_ value was estimated by inhibitory rates and calculated using Logit of probit regression in SPSS19.0.

### Analysis of the chemical composition of CFS

CFSs were centrifuged using ultrafiltration devices (1 kDa cut-off, PALL Corporation) at 650×*g* for 50 min at 4 °C. Then, the compounds of the CFSs that < 1 kDa were analysed by ultra-performance liquid chromatography (UPLC)-Q Exactive Focus-MS/MS (Thermo Scientific Co. Ltd). A C18 column (2.1 × 100 mm, 1.8 μm) was used in UPLC with 0.1% HCOOH (A) and acetonitrile (B) as mobile phase. The flow rate was as 0.3 mL/min, and the gradient was: A/B 95/5 (0 min), 10/90 (15 min), 10/90 (20 min), 95/5 (20.1 min), 95/5 (30 min). Mass spectrometry with an electrospray ionization source was used for metabolite identification. The heated capillary was maintained at 320 °C. The spray voltage was set to 3.8 kV in positive mode and 3.4 kV in negative mode. The sheath and auxiliary gases were at 25 arbs and 8 arbs, respectively. *m/z* 100 to 1500 was scanned and the three most intense peaks were collided. The compounds were identified using the Compound Discoverer 2.1.0.401 database. Peak area of an identified compound was used for relative quantification.

### Assay of cell invasion and cell migration ability

To evaluate the effect of CFS from *Lactobacillus* on CRC cell invasion and migration, Transwell™ chamber assays (Corning Incorporated Costar, USA) were used. Briefly, 3 × 10^5^ CRC cells were added into the upper chamber of each Transwell insert. For the analysis of cell invasion, the upper chambers had been coated with 200 μL of Matrigel (200 μg/mL) diluted with serum-free DMEM (cell migration did not require the Matrigel coating). The lower chamber contained 600 µL DMEM with 10% FBS (HT-29) or MEM with 20% FBS (Caco2). Cells were allowed to invade for 24 h. After that, the upper chamber was washed with phosphate-buffered saline (PBS) and carefully wiped with a cotton swab to remove excess PBS and residual cells from the upper chamber. Cells that had invaded through the Matrigel onto the bottom side of the Transwell insert were soaked in 95% ethanol and stained with crystal violet [42]. Finally, the image of the invaded cells was viewed using ToupView 3.7, and then quantified by the method of Escamilla [34]. The analysis was performed in triplicate.

### Analysis of gene expression

The impact of *Lactobacillus* CFS on the expression of *MMP2*, *MMP9*, and *VEGF* in CRC cells was analysed using SYBR Green Real-Time PCR. Briefly, CRC cells were plated on a 6 well tissue culture plate and incubated for 4 h with 3 mL CFS from 24 h cultures of strains GG, M3, or YYC-3 (pH adjusted to 7.2 using 1 M NaOH). Afterwards, cells were washed twice with PBS (pH 7.2). For cell harvest, 1 mL of PBS was added into each well and the wells were scraped with a cell scraper (Corning Incorporated Costar). Cells were collected into sterile 1.5 mL tubes, and centrifuged at 180×*g* for 2 min. The tubes and cell pellets were immediately placed into liquid nitrogen for quick-freezing. RNA was extracted from each sample according to a previously described method [43]. The quality and quantity of the extracted RNA was evaluated using a NanoDrop® ND-2000 spectrophotometer (Thermos, USA) and 1.5% modified agarose gel electrophoresis. For cDNA synthesis, PrimeScript™ RT reagent kit with gDNA Eraser was used to synthesize cDNA from the mRNA, 20 μL reverse transcription system contains 1 μg RNA. Agarose gel electrophoresis was used to confirm the synthesis of cDNA. The relative gene expression was analysed by RT-PCR, using *GAPDH* as a reference gene (primers are listed in Table 2). According to the manufacturer’s instructions, 2 μL of cDNA was added into an 18 μL reaction mixture containing 10 μM primers and 2 × Master Mix. An ABI 7500 instrument (Applied Biosystems, USA) was used to perform the RT-PCR. The amplification reaction was set as 30 s at 95 °C for 40 cycles, followed by 10 s at 95 °C, 60 °C for 60 s, 95 °C for 15 s, and then increasing the temperature from 60 °C to 99 °C to establish the melt curve of PCR products. Each sample was evaluated in triplicate. Data were analysed using the 2^−△△CT^ method by ABI7500 SD2.3 software.

**Table 2:** Primer sequences

Gene name	Forward primer (5ʹ–3ʹ)	Reverse primer (5ʹ–3ʹ)	Product size (bp)
*MMP2*	AATGCCATCCCCGATAACCT	CACGCTCTTCAGACTTTGGTTC	114
*MMP9*	GAGCACGGAGACGGGTATC	ACTCGTCATCGTCGAAATGG	106
*VEGF-A*	CAGATTATGCGGATCAAACCTCACC	CACAGGGAACGCTCCAGGACTTAT	190
*GAPDH*	CACCCACTCCTCCACCTTTGA	TCTCTCTTCCTCTTGTGCTCTTGC	188

### Analysis of protein level

The impact of Lactobacillus CFS on protein level of MMP2, MMP9, and VEGF was explored using western blot analysis. CRC cells were treated and collected according to the method described in section 'Analysis of gene expression'. After sample collection, cells were lysed for 20 min in RIPA buffer supplemented with 1 mM PMSF and protease inhibitor cocktail tablet (Roche) on ice. Then the lysed cells were centrifuged at 1300×*g* for 20 min at 4 °C, and the protein supernatants were collected. Moreover, the protein samples were homogenized by electric tissue homogenizer (TianGen BioTEC, OSE-Y30, China) with the speed of 1500×*g* for 10 s, and then homogenizing three times at 10 s intervals. After homogenization, the samples were incubated on ice for 20 min and centrifuged at 4 °C, 1300×*g* for 20 min, followed by collecting the supernatant. Furthermore, BCA protein assay kit (BioDee BioTech, China) was used to analyse the concentration of the protein and then the protein concentration was adjusted by RIPA, and the final concentration of the sample was 4 mg/mL after adding 5 × reduction sample buffer. Protein supernatants were analysed by 12% sodium dodecyl sulphate–polyacrylamide gel electrophoresis (SDS-PAGE). Proteins were transferred onto nitrocellulose membranes (Millipore, USA) by a wet transfer method at 300 mA for 120 min. After protein transfer, the membranes were blocked in 5% skim milk-TBST, and then shaken gently at room temperature for 30 min. Primary antibodies (rabbit anti-human IgG, obtained from Abcam, Cambridge, UK), including MMP2 (1:2000 dilution), MMP9 (1:2000), and VEGFA (1:2000) were added to the membranes, then retained for 10 min at room temperature, then at 4 °C. After 24 h, membranes were washed five times using TBST (3 min for each wash). Secondary antibody (goat anti-rabbit IgG, horseradish peroxidase-conjugated) obtained from TDY Biotech Co., Ltd., China was diluted 10,000 times with skim milk/TBST. Membranes were incubated with secondary antibody at room temperature for 40 min with gentle shaking. Electrochemical luminescence reagents were added to the membrane, reacted for 3–5 min, and autoradiography film was exposed to the membrane for 10 s to 5 min. Finally, the film was developed for 2 min, and fixed. Protein band intensities were analysed using Image-J software and the housekeeping protein was β-actin.

### Analysis of secretion level by ELISA kit

EliKine™ Human MMP2 and MMP9 ELISA kit (Abbkine), and Human VEGFA ELISA kit (Abcam) were used to analyse the inhibitory ability of Lactobacillus CFSs on the secretion level of MMP2, MMP9, and VEGFA. In brief, 3 × 10^5^ cells were cultured in six well plates, then samples were added and incubated for 24 h. After that, the cell supernatants were collected from each group, and the contents of these proteins were analysed according to the manufacturer’s instruction. In briefly, the specific antibody for MMP2 and MMP9 were coated on a 96 well plate. After reaction that the colour from blue to yellow in the wells, protein levels were measure using spectrophotometer (TECAN, Spark 20 M, Switzerland) and read at 450 nm.

### Assessment of MMP-2 and MMP-9 activities using gelatine zymography

MMP gelatine zymography electrophoresis analysis kit (Genmed Scientifics Inc, USA) was carried out to assess the inhibitory effect of CFSs on MMP2 and MMP9 activities. The sample was prepared as described by Escamilla et al. [34]. BCA protein assay kit (BioDee BioTech, China) was used to analyse the concentration of the protein. Then, extracellular MMP2 and MMP9 activities were tested according to the manufacturer’s instruction. Proteins were separated by 10% polyacrylamide gels of SDS-PAGE. Samples with loading buffer at 3:1 ratio for 20 μL were added in each well, and running electrophoresis at 125 V for 1.5 h. After that, the gel was washed in renaturation solution for 1 h, followed by digestion for shaking at 37 °C for 24 h. After staining and decolorization, using the termination solution to stop the reaction. Their activities were assessed by the quantification of the bands. The bands images were viewed using Clinx Genosens Capture program, and then quantified using ImageJ system.

### Statistical analysis

All data in this experiment are expressed as mean ± standard deviation of triplicate values. Data were analysed by Turkey test of One-way analysis of variance (ANOVA) in SPSS 19.0 software. *P*-value less than 0.05 was considered statistically significant.

## Supplementary information

**Additional file 1: Figure S1.** The images of cell migration. All CFS treatments inhibited cell migration compared with negative controls at the concentration of 80 μL/mL (untreated group is the negative control and the 5-FU treatment group is the positive control).

## Data Availability

The supplement data are availability from the corresponding author if required.
