# Supplementary material for: Metabolite secretions of Lactobacillus plantarum YYC-3 may inhibit colon cancer cell metastasis by suppressing the VEGF-MMP2/9 signaling pathway
Source: Microb Cell Fact. 2020 Nov 23;19:213. doi: 10.1186/s12934-020-01466-2 (PMC7684877; doi:10.1186/s12934-020-01466-2)
Supplement: Supplementary file 1 — Additional file 1: Figure S1. The images of cell migration. All CFS treatments inhibited cell migration compared with negative controls at the concentration of 80 μL/mL (untreated group is the negative control and the 5-FU treatment group is the positive control). [file 12934_2020_1466_MOESM1_ESM.docx]

**Additional file**


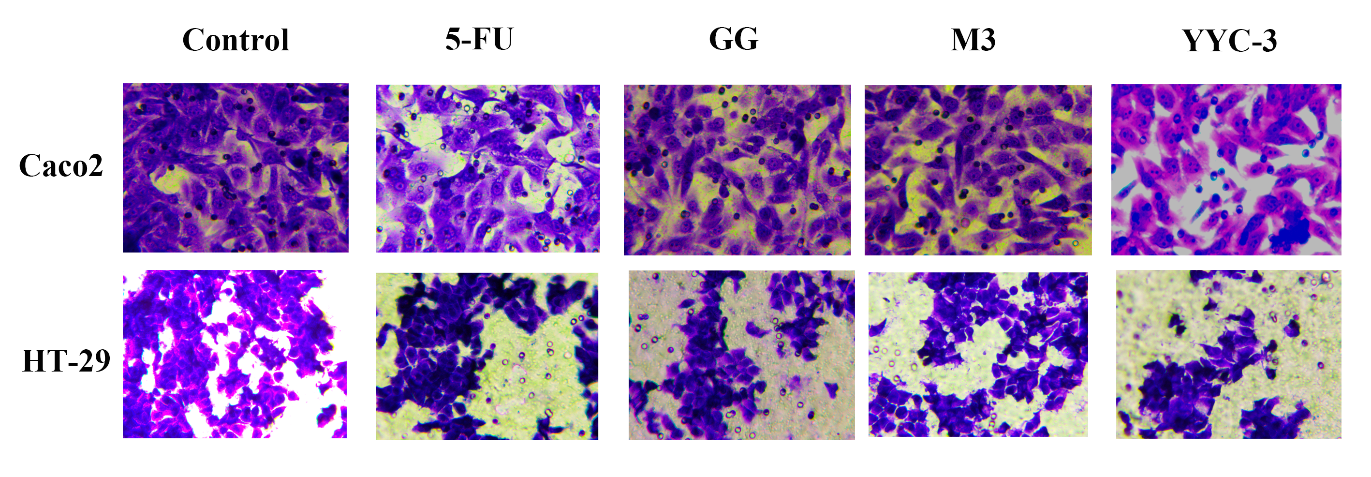


**Figure S1. The images of cell migration.** All CFS treatments inhibited cell migration compared with negative controls at the concentration of 80 ul/ml (untreated group is the negative control and the 5-FU treatment group is the positive control).
